# Factors associated with mosquito pool positivity and the characterization of the West Nile viruses found within Louisiana during 2007

**DOI:** 10.1186/1743-422X-7-139

**Published:** 2010-06-25

**Authors:** Rebecca C Christofferson, Alma F Roy, Christopher N Mores

**Affiliations:** 1 Department of Pathobiological Sciences, School of Veterinary Medicine, Louisiana State University, Baton Rouge, Louisiana, USA; 2 Louisiana Animal Disease Diagnostic Laboratory, Louisiana State University, Baton Rouge, Louisiana, USA

## Abstract

**Background:**

West Nile virus (WNV) is an arbovirus of public health importance in the genus *Flavivirus*, a group of positive sense RNA viruses. The NS3 gene has a high level of substitutions and is phylogenetically informative. Likewise, substitutions in the envelope region have been postulated to enable viruses to subvert immune responses. Analysis of these genes among isolates from positive mosquitoes collected in Louisiana illustrates the variation present in the regions and provides improved insight to a phylogenetic model. Employing a GIS eco-regionalization method, we hypothesized that WNV pool positivity was correlated with regional environmental characteristics. Further, we postulated that the phylogenetic delineations would be associated with variations in regional environmental conditions.

**Results:**

Type of regional land cover was a significant effect (p < 0.0001) in the positive pool prediction, indicating that there is an ecological component driving WNV activity. Additionally, month of collection was significant (p < 0.0001); and thus there is a temporal component that contributes to the probability of getting a positive mosquito pool. All virus isolates are of the WNV 2002 lineage. There appears to be some diversity within both forested and wetland areas; and the possibility of a distinct clade in the wetland samples.

**Conclusions:**

The phylogenetic analysis shows that there has been no reversion in Louisiana from the 2002 lineage which replaced the originally introduced strain. Our pool positivity model serves as a basis for future testing, and could direct mosquito control and surveillance efforts. Understanding how land cover and regional ecology effects mosquito pool positivity will greatly help focus mosquito abatement efforts. This would especially help in areas where abatement programs are limited due to either funding or man power. Moreover, understanding how regional environments drive phylogenetic variation will lead to a greater understanding of the interactions between ecology and disease prevalence.

## Background

West Nile Virus (WNV) is the most widely distributed arbovirus in the world, occurring on all continents save Antarctica [1]. Its lack of vector specificity compared to other arboviruses has allowed it to use a wide variety of mosquito species in its enzootic cycles [2]. WNV was introduced into the United States in 1999 and from its entry point of New York City it spread across the continental United States. Phylogenetic evidence traced this strain to a similar strain isolated in Israel in 1998 [2]. In 2001 a new WNV strain appeared. In 2002, this genotype became the dominant WN02 strain that was significantly associated with an increase in numbers of human morbidity and mortality cases in the US. In fact, the number of deaths from 1999-2001 were significantly less than the number of deaths in 2002 alone, though whether this association is due to direct virulence in humans or an indirect result of the virulence in birds remains unclear [1,3,4].

WNV is a member of the genus *Flavivirus*, a group of positive sense RNA viruses. The genome is composed of a single open reading frame that produces ten viral proteins: three structural proteins (capsid C, membrane prM/M, envelope E) and seven non-structural proteins (NS1, NS2A, NS2B, NS3 NS4A, NS4B, and NS5) [5]. The NS3 gene plays an important role in the replication of the virus, encoding a protein with four functions: a serine protease, a nucleoside triphosphatase, an RNA 5'triphosphatase, and a helicase [5]. Phylogenetic analyses of WNV have most commonly utilized differences in the envelope protein, but the capsid, prM protein, and non-structural proteins have also been informative [4,6-12]. Analyses done on complete genomes have given similar results to trees made from prM and envelope proteins [4]. To determine the genetic variability in Louisiana, the envelope coding and NS3 coding regions were analyzed. The NS3 gene has a high level of substitution and is phylogenetically informative [11] and mutations in the envelope region have been postulated to enable viruses to subvert immune responses [13]. Analysis of these genes would illustrate the variation present in Louisiana as well as provide improved insight for our phylogenetic model.

Many phylogenetic studies have been geographically focused [6,8,14,15]. Geographic Information Systems (GIS) based "region" classifications were successfully used to model WNV transmission risk in humans in northeast Ohio where local environmental features to model transmission [16]. Employing a similar eco-regionalization method, we hypothesized that WNV pool positivity was correlated with regional environmental characteristics. Further, we postulated that the phylogenetic delineations would be associated with variations in regional environmental conditions.

## Results

### Positive Pool Predictors

**Figure 1:**
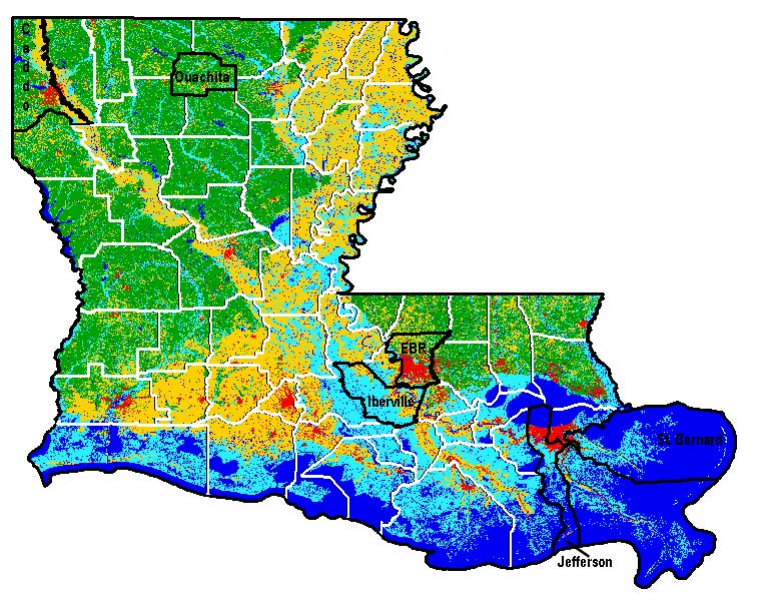
**Louisiana Landcover**. Louisiana GIS Digital Map, May 2007 shows the land cover of Louisiana. Pertinent parishes are labelled with their parish names.

The data used in the modelling study were from several parishes in Louisiana during 2007. There were 611 positives reported by the Louisiana Animal Disease Diagnostic Laboratory, 165 in our target parishes. Classification regions were constructed based on land cover data from the Louisiana GIS Digital Map, May 2007(Figure 1). The land cover of a parish was determined by the majority rule. Ouachita and Caddo parishes are, for example, 50-75% forest lands; East Baton Rouge is a majority developed area comprised mostly of the urbanized capital of Baton Rouge [17]. Iberville Parish is classified as wetlands, defined as low lying areas saturated with moisture.

Before building the WNV model, we tested several factors via multinomial regression to determine whether these were significant predictors of the mosquito species found, regardless of WNV positivity. Interestingly, no factors (land cover, month) were significant for the 2007 data. Therefore, it was determined that no noteworthy correlation existed between these variables and thus no colinearity issues would arise by inclusion of all variables in the model.

The model originally included the effects of month, mosquito genus and species; but through a stepwise selection procedure, the variable species was not significant at the alpha = 0.05 level and thus eliminated from the model. This is likely due to the overwhelming number of *Culex quinquefasciatus*, which comprised over 61% of the total pools (N_total _= 3246) and 88% of the positive pools (Table 1). This is consistent with earlier studies where *Cx. quinquefasciatus *was found to be the most abundant and likely epizootic vector for the virus [18,19]. In addition, all interactions were not significant and therefore removed from the model.

**Table 1:** Species in Positive Mosquito Pools

Genus	Species	Number of positive pools
*Aedes*	*albopictus*	12
	*triserriatus*	1
	*vexans*	5
*Anopheles*	*quadrimaculatus*	1
*Culex*	*coronator*	2
	*erraticus*	3
	*nigrapalpus*	1
	*quinquefasciatus*	129
	*restuans*	1
	*salinarus*	1
	*tarsalis*	1
*Mansonia*	*titillans*	2
*Psorophora*	*columbiae*	3
	*Ferox*	2
	*howardii*	1

Mosquito pools by species positive for **WNV **in 2007 from the four target parishes.

**Figure 2:**
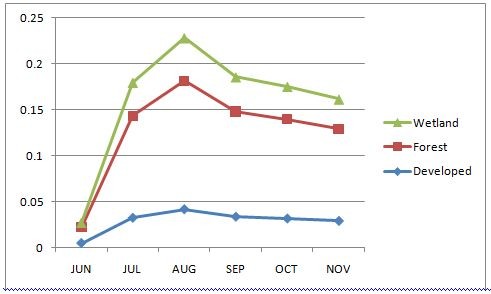
**Positive Pool probability over months by land cover**. Predicted probability of a positive mosquito pool between June and November 2007 as predicted by land cover. Included are reference strains used in Ebel, et. al. [15]

Locations of isolates were coded according to land cover. The model included land cover, genus, and month. Type of regional land cover was a significant effect (p < 0.0001) indicating that there is an ecological component driving WNV activity. Additionally, month was significant (p < 0.0001); and thus there is a temporal component that contributes to the probability of getting a positive mosquito pool. This trend can be seen in figure 2.

The pair wise differences in least squares means are given in table 2 with Tukey adjusted p-values showing where the significant differences lay. Forested lands are clearly more likely to have a positive mosquito pool as compared to developed areas and wetlands. Similarly, August appears to be the month where a positive mosquito pool is more likely. August and July are eight and six times more likely to see a positive mosquito pool than June, respectively. Interestingly, August and July are not significantly different from the months of September-November. All months are significantly different from June, which is the least likely month during the accepted transmission season to have a positive pool; the odds of a positive pool is over seven times more likely in July than June. The overall trends of land cover and month are shown together in Figure 2.

**Table 2:** Genetic Distances for 2007 Samples

	Within	Between
	
Louisiana	.29104	.4751
Outside LA	.56137	
Forest	.55029	.65608
Wetlands	.35147	

Genetic distances of the 2007 samples from Mega 4.0 show more variability within Louisiana than when Louisiana is compared to the rest of North American isolates.

### Phylogenetic Analysis

**Figure 3:**
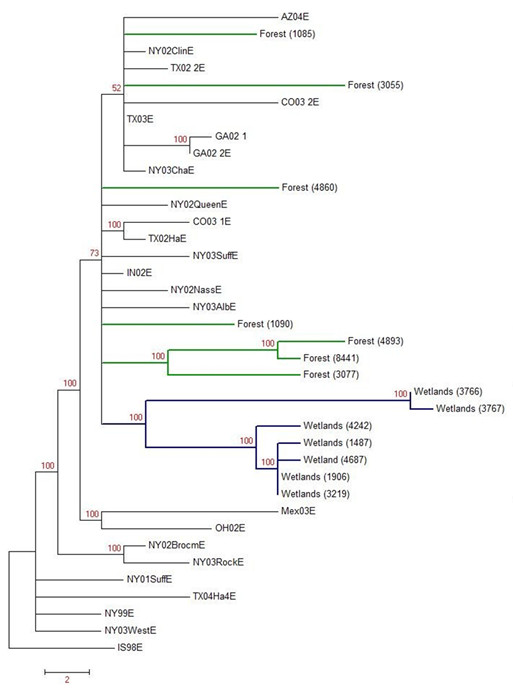
**Maximum Parsimony Phylogenetic Tree **- Phylogenetic tree based on 1000 bootstraps of the maximum parsimony analysis of the WNV E and NS3 contiguous sequence. Branches indicating Louisiana isolates are coded by eco-region with isolate ID in parentheses. Other sequences included in this phylogeny include the following GenBank accession numbers: [Genbank:AF260967, Genbank:DQ164201, Genbank:DQ164195, Genbank:DQ164191, Genbank:DQ164190, Genbank:DQ164189, Genbank:DQ164203, Genbank:DQ164204, Genbank:DQ164199, Genbank:AY712948, Genbank:DQ164194, Genbank:DQ164192, Genbank:AY033389, Genbank:DQ164196, Genbank:DQ164197, Genbank:DQ164193, Genbank:DQ164205, Genbank:AY963775, Genbank:DQ164200, Genbank:DQ164198, Genbank:DQ164202, Genbank:DQ164188, Genbank:DQ164187, Genbank:DQ164186].

The phylogenetic tree resulting from the contiguous segment comprised of the envelope and NS3 sequences is shown in figure 3. All samples are, unsurprisingly, of the 2002 lineage. However, there appears to be some diversity within both forested and wetland areas. Particularly, there is a possible small sub-group with samples 4893, 8441, and 3077. The samples from the wetlands show the possibility of a distinctive group; in particular, sample numbers 3766 and 3767. There is a clear delineation between forest and wetland samples based on two nucleotide substitutions in the NS3 gene: an adenine to guanine at positions 5760, and a cytosine to uracil at position 6324., Samples were grouped as follows: Israel 1998 as the root; the wetlands as a group; a small grouping of forest samples; a NY99 group; and the remaining were grouped together as representative of the North American 2002 clade. The between and within distances were computed according to the Jukes-Cantor model (table 3). Genetic diversity within Louisiana was modest when compared to isolates from a wide geographic range. The genetic distances within the eco-regions (forest vs. wetlands) in Louisiana were greater than the distances comparing Louisiana and those strains from outside of Louisiana. Table 4 identifies sample origins, classifications, and accession numbers.

**Table 3:** Origin and classification of sequenced isolates

Land Cover	ID Number	Parish	Accession Number (E/N)
Wetlands	3766	St. Bernard	HM538823/HM538811
	3767	St. Bernard	HM538824/HM538812
	3219	St. Bernard	HM538829/HM538807
	4242	Iberville	HM538825/HM538810
	1487	Jefferson	HM538827/HM538806
	4687	Jefferson	HM538826/HM538808
	1906	Jefferson	HM538828/HM538809

Forrest	3055	Caddo	HM538821/HM538813
	1085	Caddo	HM538817/HM538805
	4860	Ouachita	HM538822/HM538816
	4893	Ouachita	HM538818/HM538803
	8441	Ouachita	HM538819/HM538804
	3077	Ouachita	HM538820/HM538814

The isolates that were successfully sequenced are listed by land cover classification. The parish where each was collected is identified and accession numbers for E (envelope) and N (NS3) sequences are given as well.

**Table 4:** Differences of Least Square Means

Effects		Odds Ratio	Adjusted P-value
Developed	Forest	0.279	<.0001
Wetland	Forest	0.346	0.0076

July	June	6.65	0.0006
August	June	8.98	<.0001
September	June	7.09	0.0001
October	June	6.75	<.0001

The differences in the least square means reported as odds ratios with Tukey adjusted p-values show significant differences between types of land cover and month.

## Discussion

In some locations, WNV positivity in mosquito pools serves as a predictor for human cases [14]. Our findings that the month of collection and eco-region were significant predictors of positive pools suggest that ecological and temporal factors influence WNV activity and can assist the public health sector predict or prevent cases of human WNV infection. Mosquito abatement programs are operated on a parish-wide basis, so any useful model would ideally work for the parish as a whole. Therefore, looking at the ecology of the parish as a whole- and even grouping parishes into ecological regions or types- will assist in determining a robust model as we did in our pool positivity model, can be used at the very least, on a parish level. Ideally, a model would serve the state as a whole, focusing on regional activity.

Phylogenetic analysis will also help us better understand how the changes in the genome may lead to a change in virulence. It is interesting that the statistical model predicts positive pools in forested areas while the phylogenetic study shows more diversity in the wetland areas. It is also interesting that there is more genetic diversity within Louisiana than without. That is, the diversity when comparing between the wetlands and forests within Louisiana is greater than the diversity when comparing the Louisiana isolates and those outside of the state. This suggests that WNV evolution is not as constrained in Louisiana as compared to other locations as has been suggested by others [20]. Further, some component of eco-regions could have an important role in diversification. This could be due to a difference in bird populations found in each of these ecologies, or it could be a function of extrinsic, microhabitat conditions, such as temperature or humidity which could exert a selection pressure on the viruses.

## Conclusions

The phylogenetic analysis shows that there has been no reversion in Louisiana from the 2002 lineage which replaced the originally introduced strain. Our statistical model serves as a basis for future testing, directing mosquito control efforts and surveillance programs. Though we believe our findings to be a significant start to a potentially long term project, it was not without its pitfalls. For example, there is a confounding factor of spatial clustering of the ecologies tested. However, if the spatial component was the only source of diversity, thus discounting the ecological diversity, we would not expect that the phylogenetic signature of the Southern Louisiana strains should be markedly different in topology than the Northern Louisiana strains. The fact that the wetland strains formed a distinct monophyletic group as compared to the forest strains, which were characterized by a lack of phylogenetic structure, suggests the diversity seen within these regions is the result of some other, perhaps ecological, characteristic.

Additionally, the specificity of the land cover classification needs to be more precise. This land cover generalization to parish is a good start to determine if further investigation is garnered, and here we show that it is. Understanding how land cover and regional ecology effects mosquito pool positivity will help focus mosquito abatement efforts. This would especially help in areas where abatement programs are limited due to either funding or man power. Moreover, understanding how regional environments drive phylogenetic variation will lead to a greater understanding of the interactions between ecology and disease prevalence.

## Methods

### Samples

According to the mosquito surveillance data, there are two primary species of mosquitoes that serve as possible WNV vectors to people in Louisiana: *Culex quinquefasciatus *and *Aedes albopictus*. There were twenty six species from eight genera captured and submitted for testing. Of these, fifteen species were found to be positive for WNV (Table 1).

### Mosquito Trap Sites and Field Collection of Mosquitoes

Data was provided by parish mosquito control departments. Each parish operates independently with its own trapping protocols and methods. Not all parishes actively sampled throughout the year due to considerations of the local mosquito activity levels. We therefore analyzed the months that all target parishes had in common (June-November), which captured the majority of the WNV transmission period [21].

Mosquitoes were sexed and the females were pooled according to genus and species; that is, one pool consisted of a single species. The pools were then homogenized and submitted to the Louisiana Animal Disease Diagnostic Laboratory (LADDL) at the Louisiana State University (LSU) School of Veterinary Medicine (SVM). LADDL is the state testing facility for mosquito pools for all parishes, so criteria for positive pools is the same across all parishes.

### Virus Detection and Sequencing

Pools were obtained from the LADDL at the LSU SVM. Viral RNA was extracted from 140 μl of the supernatant from the mosquito pool homogenate using the QIAmp Viral RNA Extraction Kit following manufacturer's instructions (Qiagen, Valencia, CA). One microliter of the extracted viral RNA suspension was used as template for the reverse-trascriptase polymerase chain reaction (RT-PCR) using Superscript™ III RT-PCR kit (Invitrogen, Carlsbad, CA) with the previously described protocol [22]. Upon confirmation of the presence of amplified viral DNA by gel electrophoresis, the remaining sample was cleaned using Qiagen PCR Cleanup kit following manufacturer's instructions. Cleaned DNA was then sent to the Gene Probes and Expression Systems Laboratories of the Division of Biotechnology and Molecular Medicine at the Louisiana State University School of Veterinary Medicine for sequencing. Sequencing was performed on a Beckman Coulter 8800 (Pasadena, CA) using the manufacturer's reagents and methods.

### Statistical Analysis

SAS version 9.1.3 was used to code the data as a binary response, where a mosquito pool that was positive for was coded as '1,' while the negative pools were coded as '0.' The probabilities reported are the probabilities of the event = 1 (WNV positive). A confidence level of 95% was used for all tests; a stepwise selection process was invoked to cull out non-significant effects from the model. PROC GLIMMIX with a binary distribution specified was used to construct the model and obtain the odds ratios, as well as to obtain differences in least squares means for the effects of parish and month. PROC GLIMMIX is a useful alternative to PROC LOGISTIC when wanting to obtain differences in least squares and/or modeling random effects, which is not easily done in PROC LOGISTIC. Predicted means and odds ratios are the same between the two procedures. Confidence intervals and tests for significance will vary when random effects are included the model in PROC GLIMMIX, but there were no random effects modelled here [23].

### Phylogenetic Analysis

The complete envelope (E) and non-structural protein 3 (NS3) genes were aligned separately for all successfully recovered isolates as well as 24 reference strains, which represented the Israel 1998 strain and isolates spanning the contiguous United States and one Mexican isolate [15]. Alignments and the creation of the E-NS3 contigs were done using Vector NTI software and exported to GeneDoc for trimming. Alignments were imported into MEGA 4 and converted to MEGA format. Bootstrap analysis (n = 1000) using maximum parsimony was performed and a tree produced. As the topologies of the E and NS3 phylogenies were the same, our tree represents a contiguous sequence of these two genes. The topology tree was collapsed with a node confidence of 70%. Genetic distances and means were also obtained using Mega 4 [24]. Not all samples in the statistical model were available for the phylogenetic analysis.

## Competing interests

The authors declare that they have no competing interests.

## Authors' contributions

RCC contributed to the molecular work, statistical analysis. RCC and CNM contributed to the writing and preparation of the manuscript and conceptualization of the model. CNM and AFR provided logistical support and mentoring. All authors have read and approved this manuscript.
